# Expression and prognostic value of cholesterol homeostasis genes in hepatocellular carcinoma: A cohort study based on TCGA

**DOI:** 10.1097/MD.0000000000048547

**Published:** 2026-05-22

**Authors:** Chongyu Ding, Jianghua Huo, Ya-Qian Xu, Hui Zhang, Yulu Gong, Darong Hao, Dan Meng

**Affiliations:** a School of Global Health, Chinese Centre for Tropical Diseases Research, Shanghai Jiao Tong University School of Medicine, Shanghai, PR China; b School of Public Health and Management, Jiangsu Medical College, Jiangsu, PR China; c Community Health Service Center of Haiwan Town, Shanghai, PR China.

**Keywords:** alcohol dehydrogenase 4 (ADH4), cholesterol homeostasis genes, choline kinase alpha (CHKA), fatty acid-binding protein 5 (FABP5), hepatocellular carcinoma, squalene epoxidase (SQLE)

## Abstract

Cholesterol metabolism is critical in cancer biology, yet its role in hepatocellular carcinoma (HCC) remains poorly understood. We investigated the expression and prognostic significance of cholesterol homeostasis genes in HCC to identify potential biomarkers for risk stratification. Using data from The Cancer Genome Atlas, we identified differentially expressed cholesterol-related genes (logFC ≥ 1.5, false discovery rate < 0.05), intersecting them with genes annotated in the Molecular Signatures Database. Prognostic biomarkers were evaluated using univariate and multivariate Cox regression. A predictive model was constructed to assess survival outcomes. Cholesterol homeostasis genes, particularly fatty acid-binding protein 5 (FABP5) and alcohol dehydrogenase 4 (ADH4), are key prognostic biomarkers in HCC. The proposed model provides a novel approach for predicting outcomes and may inform therapeutic strategies. Further studies are needed to validate these findings and explore their clinical utility. Nine cholesterol homeostasis genes were differentially expressed, including 6 upregulated (choline kinase alpha, FABP5, fatty acid synthase, farnesyl diphosphate synthase, mevalonate diphosphate decarboxylase, and squalene epoxidase) and 3 downregulated (ADH4, activating transcription factor 5, and arginine vasopressin receptor 1A). FABP5 and ADH4 emerged as independent prognostic factors: high FABP5 expression was associated with poor prognosis, while high ADH4 expression predicted improved survival. A prognostic model integrating FABP5 and ADH4 and clinical characteristics achieved a concordance index of 0.683, effectively stratifying patients into high-risk and low-risk groups (χ^2^ = 20.60, *P* < .001) with median survival times of 1135 and 2532 days, respectively.

## 1. Introduction

Hepatocellular carcinoma (HCC) is the sixth most common cancer globally, with an incidence rate of 4.7%, and the third leading cause of cancer-related deaths, accounting for 8.3% of all cancer mortalities.^[1]^ Over 80% of cases occur in low- and middle-income countries, particularly in East Asia and Sub-Saharan Africa.^[2]^ These alarming statistics underscore the urgent need for deeper insights into the molecular mechanisms underlying HCC to improve diagnosis and treatment.

Cholesterol homeostasis, governed by genes involved in biosynthesis, uptake, excretion, and storage, is essential for cellular and systemic function.^[3]^ Cancer-associated metabolic reprogramming disrupts cholesterol homeostasis, leading to increased cholesterol uptake and synthesis, reduced efflux, and altered lipid storage, fueling tumor growth.^[4–7]^ In HCC, deregulation of cholesterol homeostasis genes promotes abnormal cholesterol accumulation and the formation of metabolic byproducts, driving tumor progression.^[5,6]^

As a molecularly heterogeneous malignancy, HCC’s biological behavior is closely linked to its molecular characteristics.^[8]^ Understanding the expression and prognostic significance of cholesterol homeostasis genes is critical for elucidating the biology of HCC and identifying therapeutic targets. Although prior studies have examined cholesterol metabolism in cancer, the roles and prognostic relevance of these genes in HCC remain poorly defined. This study addresses this gap by employing bioinformatics to investigate the expression of genes involved in cholesterol homeostasis and their prognostic implications in HCC.

## 2. Materials and methods

### 2.1. Data collection

RNA-sequencing data and clinical information for 374 HCC samples and 50 tumor-adjacent normal liver tissue samples were retrieved from The Cancer Genome Atlas (TCGA) database (https://portal.gdc.cancer.gov/). After excluding subjects without survival time, 370 HCC samples from TCGA were included in this study for further analysis and the clinical characteristics are shown in Table S1. Ethical approval was not necessary because TCGA is publicly available for researchers to publish relevant articles. A curated set of 74 cholesterol homeostasis-related genes was obtained from the Molecular Signatures Database (MSigDB), a widely recognized resource for cancer and metabolic research.^[9]^ Liver cancer data from the International Cancer Genome Consortium (ICGC) datasets, which were downloaded from the Sangerbox platform, were used as the validated population in this study.^[10]^ The ICGC datasets are publicly available and includes genomic alterations (e.g., mutations and copy number variations) and clinical information for patients with liver cancer.

### 2.2. Identification of differentially expressed cholesterol homeostasis genes

Firstly, mRNA expression profiles were annotated using Refseq transcript ID and Ensembl gene ID, Subsequently, differentially expressed genes (DEGs) between tumor and normal tissues were identified using R, with filtering criteria of |log_2_ Fold Change| ≥ 1.5 and a false discovery rate < 0.05. Finally, DEGs overlapping with the MSigDB cholesterol homeostasis gene set were visualized using a Venn diagram. Expression differences between tumor and normal tissues were analyzed using the Wilcoxon rank-sum test.

### 2.3. Cox regression analysis of prognostic genes

Univariate Cox regression was conducted to assess the associations between the expression of DEGs and overall survival in 370 HCC patients with complete survival data. Genes with *P* < .1 were considered as survival related genes and further filtered by multivariate Cox regression analysis, adjusting for age, sex, TNM stage, and WHO grade. Genes with *P* < .05 in the multivariate Cox regression analysis were considered as independent prognostic genes and their expressions were analyzed across clinical subgroups.

### 2.4. Survival and risk score analysis

HCC patients were stratified into high- and low-expression groups according to the median expression level of each significant independent prognostic genes. Kaplan–Meier survival curves were plotted and log-rank tests were used to compare survival differences between 2 groups. A risk score formula was derived from the multivariate Cox model according to the expression levels of the identified independent prognostic genes by summing the normalized expression of each gene weighted by the corresponding regression coefficient.^[11]^ Then the risk score was calculated for each patient and used to classify patients into high- and low-risk groups according to the median risk scores. Survival differences between the different risk groups were assessed using the Kaplan–Meier method. The expression and prognostic value of these independent prognostic genes were further validated using liver cancer subjects from the ICGC database.

### 2.5. Construction and evaluation of the prognostic prediction nomogram

A prognostic nomogram was developed based on the expression levels of the identified independent prognostic genes and clinical information to predict the overall survival of HCC patients in TCGA. To determine the optimal combination of predictors, a full model and bidirectional stepwise model were applied. The model with the lowest Akaike information criterion value was selected as the final prediction model and the risk score for each patient was calculated as a linear combination of the selected variables weighted by their respective regression coefficients from the final Cox model. Predictive accuracy was evaluated using the concordance index (C-index).

### 2.6. Statistical analysis

Statistical analyses were performed using R software (version 4.2). Gene expression comparisons were conducted using the Wilcoxon rank-sum test, and survival analyses were performed using Kaplan–Meier curves and log-rank tests. Statistical significance was defined as *P* < .05.

## 3. Results

### 3.1. Differential expression analysis of cholesterol homeostasis genes

**Figure 1.**
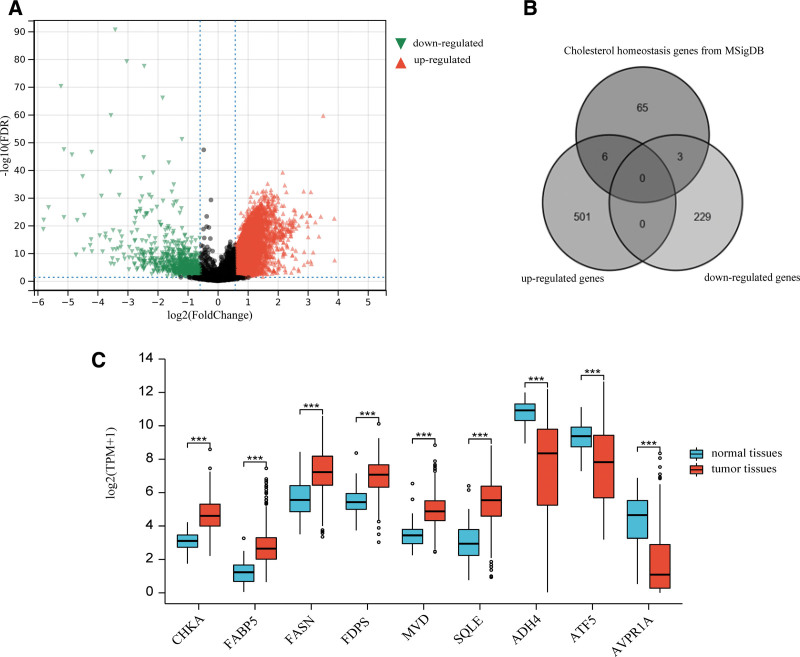
The results of analysis of differentially expressed genes. (A) Volcano plot of differentially expressed genes in TCGA_LIHC; (B) Venn diagram of cholesterol homeostasis genes and differentially expressed genes; and (C) expression of differentially expressed cholesterol homeostasis genes. ADH4 = alcohol dehydrogenase 4, ATF5 = activating transcription factor 5, AVPR1A = arginine vasopressin receptor 1A, CHKA = choline kinase alpha, FABP5 = fatty acid-binding protein 5, FASN = fatty acid synthase, FDPS = farnesyl diphosphate synthase, MVD = mevalonate diphosphate decarboxylase, SQLE = squalene epoxidase.

Figure 1 illustrates the differential expression analysis of mRNA data from 374 HCC tumor samples and 50 normal liver tissue samples obtained from TCGA database. A total of 739 DEGs were identified, of which 507 were upregulated and 232 were downregulated (Fig. 1A). A Venn diagram visualizes the intersection of these DEGs with the cholesterol homeostasis gene set from the MSigDB (Fig. 1B). This analysis identified 9 differentially expressed cholesterol homeostasis genes, consisting of 6 upregulated genes (choline kinase alpha [CHKA], fatty acid-binding protein 5 [FABP5], fatty acid synthase [FASN], farnesyl diphosphate synthase [FDPS], mevalonate diphosphate decarboxylase, and squalene epoxidase [SQLE]) and 3 downregulated genes (alcohol dehydrogenase 4 [ADH4], activating transcription factor 5, and arginine vasopressin receptor 1A). The relative expression levels of these 9 genes in the tumor and normal tissues are shown in Figure 1C.

### 3.2. Prognostic significance of differentially expressed cholesterol homeostasis genes

**Figure 2.**
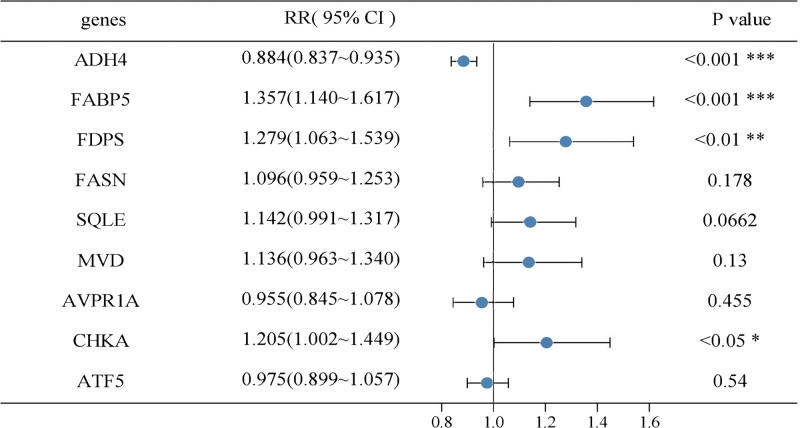
Forest plot of univariate Cox regression analysis. ADH4 = alcohol dehydrogenase 4, ATF5 = activating transcription factor 5, AVPR1A = arginine vasopressin receptor 1A, CHKA = choline kinase alpha, FABP5 = fatty acid-binding protein 5, FASN = fatty acid synthase, FDPS = farnesyl diphosphate synthase, MVD = mevalonate diphosphate decarboxylase, SQLE = squalene epoxidase.

Figure 2 presents a forest plot from the univariate Cox regression analysis conducted on 370 HCC patients with available survival data. This analysis revealed that the expression levels of FABP5, ADH4, FDPS, SQLE, and FASN were significantly associated with patient prognosis. Table 1 shows the results of the multivariate Cox regression analysis, which was adjusted for clinical factors such as age, sex, TNM stage, and WHO grade. Multivariate analysis indicated that FABP5 and ADH4 were independent prognostic factors for survival in HCC.

**Table 1:** The results of multi-variates Cox regression analysis*.

Gene	β	RR	95% CI	Z	*P*
ADH4	−0.081	0.922	0.863–0.985	−2.408	.016
FABP5	0.222	1.249	1.015–1.536	2.101	.036
FDPS	0.190	1.209	0.936–1.562	1.452	.147
SQLE	−0.033	0.967	0.780–1.200	−0.305	.761
CHKA	0.125	1.133	0.897–1.432	1.047	.295

95% CI = 95% confidence interval, ADH4 = alcohol dehydrogenase 4, CHKA = choline kinase alpha, FABP5 = fatty acid-binding protein 5, FDPS = farnesyl diphosphate synthase, RR = rate ratio, SQLE = squalene epoxidase.

* Adjusted for clinical factors including age, sex, TNM Stage, and WHO grade.

### 3.3. Clinical characteristics and survival analysis of prognostic genes

**Figure 3.**
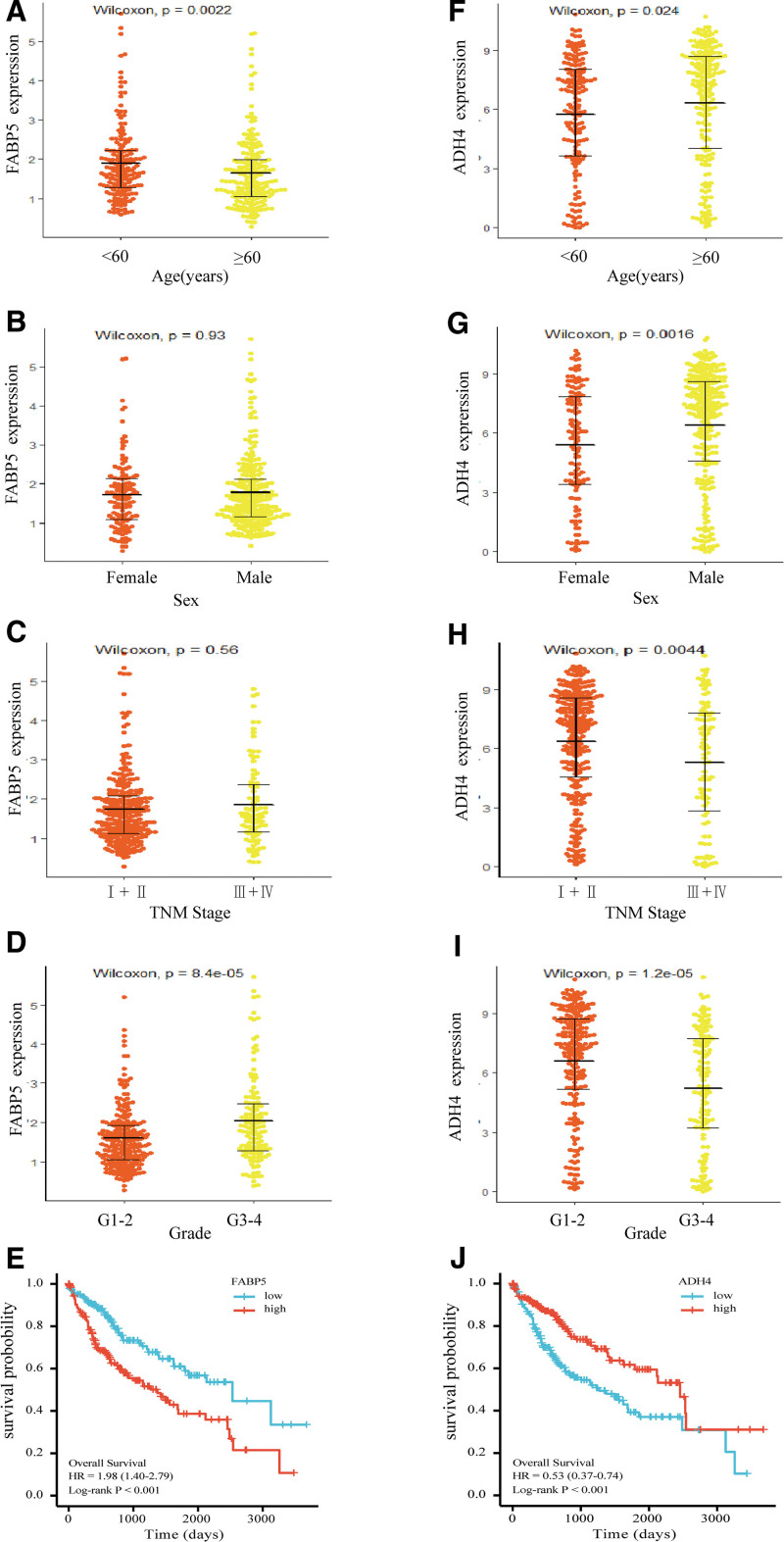
The expression of FABP5 and ADH4 genes in different clinical characteristics groups and their prognostic effect in HCCs. (A–D) The expression of FABP5 in different groups of HCC patients according to age, sex, TNM stage, and WHO grade characteristics; (F–I) the expression of ADH4 in different groups of HCC patients according to age, sex, TNM stage, and WHO grade characteristics; (E) the overall survival curves for HCC according to the expression of FABP5; and (J) the overall survival curves for HCC according to the expression of ADH4. ADH4 = alcohol dehydrogenase 4, FABP5 = fatty acid-binding protein 5, HCC = hepatocellular carcinoma.

Figure 3 shows the expression of FABP5 and ADH4 across the different clinical subgroups and their prognostic effects in HCC patients. The expression of FABP5 varied significantly across age groups (*P* = .0022) and WHO grades (*P* = .024), with no significant differences observed based on sex (*P* = .93) or TNM stage (*P* = .56; Fig. 3A). In contrast, ADH4 expression differed significantly across all clinical subgroups (*P* < .05; Fig. 3B). High FABP5 expression was associated with poorer prognosis, whereas high expression of ADH4 was associated to a better prognosis. However, although the expression patterns of ADH4 and FABP5 observed in the validation liver cancer subjects from ICGC were consistent with those found in TCGA, their prognostic significance differed. Specifically, FABP5 expression was not significantly associated with patient survival (HR = 1.799, *P* = .06), indicating a lack of prognostic value in the validation population.

### 3.4. Development of a prognostic model using FABP5, ADH4 expression, and clinical characteristic

Based on the results of the full and stepwise models incorporating FABP5 and ADH4 expression and clinical characteristics (Table S2), the stepwise model, which demonstrated superior fit performance (Akaike information criterion = 1247.55, C-index = 0.683, shown in Table S3), was ultimately selected to establish the risk score formula. The risk score formula incorporating the expression of FABP5, ADH4 gene and clinical characteristic is as follows:


$$\begin{array}{l} \begin{array}{lll} & Risk\;Score=(0.0114\times Age)+(0.9407\times I[TNMstage=T3/T4])\; & +(0.2235\times FABP5)-(0.0884\times ADH4).\; \end{array} \end{array}$$

Where I(·) is the indicator function (when TNM stage = T3/T4 is true, taking the value 1 otherwise 0). Higher risk scores indicate an increased risk of mortality.

**Figure 4.**
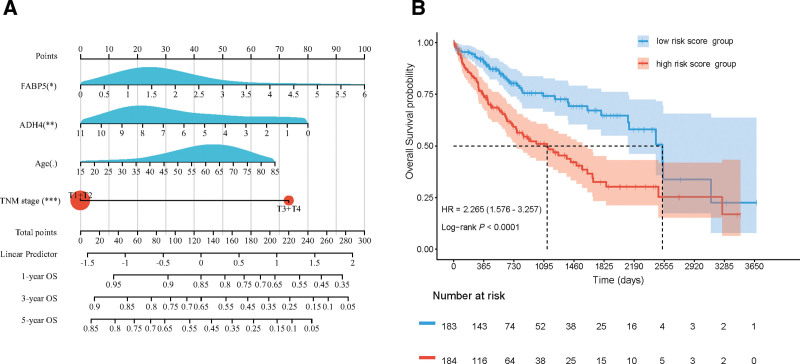
Nomograms of the prognostic prediction model and survival curves for different risk score groups. (A) Nomogram of the prognostic prediction model for hepatocellular carcinoma patients and (B) survival curves for high-risk score group and low-risk score group. ADH4 = alcohol dehydrogenase 4, FABP5 = fatty acid-binding protein 5.

Figure 4 displays the nomograms of the prognostic prediction model and survival curves for the different risk score groups. Based on the median risk score, patients were stratified into high and low-risk groups. The median survival time of the high-risk group (1135 days) was significantly lower than that of the low-risk group (2532 days; χ^2^ = 20.60, *P* < .001). Survival analysis indicated that the high-risk group had a worse prognosis, as shown in Figure 4B. The prognostic prediction model had a C-index of 0.679 (95% confidence interval: [0.627–0.732], *P* < .001), as shown in Figure 4A.

## 4. Discussion

This study provides a comprehensive analysis of cholesterol homeostasis genes in HCC, identifying 9 DEGs, including 6 upregulated genes (CHKA, FABP5, FASN, FDPS, mevalonate diphosphate decarboxylase, and SQLE) and 3 downregulated genes (ADH4, activating transcription factor 5, and arginine vasopressin receptor 1A). Among the 9 DEGs, previous studies have demonstrated that the expression of FABP5, FASN, SQLE, CHKA, FDPS and ADH4 is closely involved in the occurrence, invasion and metastasis of HCC,^[12–18]^ but the association between the expression of the other 3 genes and HCC is dearth of evidence. In our study, FABP5 and ADH4 were identified as independent prognostic factors for HCC survival. Elevated FABP5 expression was significantly associated with poor prognosis, whereas higher ADH4 expression was associates with better survival outcomes. These findings underscore the critical role of cholesterol homeostasis in HCC progression and suggest that these genes may serve as valuable prognostic biomarkers and potentially inform therapeutic strategies.

Cholesterol metabolism has emerged as a fundamental aspect of tumor biology, with accumulating evidence support its role in various cancers. Cholesterol is essential for cellular membrane structure, signaling, and energy production, which are all critical processes for cancer cell survival and proliferation. While high serum cholesterol levels are associated with an increased risk of cancers such as colorectal and prostate cancer,^[19–21]^ they exhibit an inverse correlation with liver cancer. This suggests that the relationship between cholesterol and cancer may be tissue- and context-dependent, highlighting the need for a nuanced understanding of the role of cholesterol in different cancer types.^[19]^ Furthermore, dietary cholesterol intake has been linked to an increased risk of breast cancer.^[22]^ The pro-tumorigenic effects of cholesterol are particularly evident in animal models, where a high-cholesterol diet accelerates tumor formation and metastasis.^[23–25]^ These findings suggest that changes in cholesterol metabolism, such as enhanced de novo synthesis, increased cholesterol uptake, and impaired efflux, may contribute to the elevated intracellular cholesterol levels in cancer cells. These alterations support oncogenic signaling, apoptosis evasion, and tumor cell migration, ultimately facilitating cancer progression.^[5]^

Our findings align with these previous observations. We observed increased expression of SQLE and CHKA in liver cancer tissues, corroborating earlier reports of their involvement in HCC progression.^[26]^ SQLE, a key enzyme in cholesterol biosynthesis, regulates intracellular cholesterol levels. Its overexpression in HCC, particularly in patients with nonalcoholic fatty liver disease, enhances carcinogenesis by promoting cholesterol esterification and increasing NADP^+^ levels – critical factors for tumor growth, cell proliferation, and metastasis.^[14,16]^ Moreover, SQLE’s activation of the ERK signaling pathway amplifies its oncogenic effects, making SQLE a potential therapeutic target. Inhibitors of SQLE, such as Terbinafine, have shown efficacy in reducing liver cancer growth, suggesting that targeting this enzyme could be a promising therapeutic strategy for HCC.^[14]^

CHKA, involved in phosphatidylcholine biosynthesis, is another key regulator of cholesterol homeostasis in cancer cells. Elevated CHKA expression in HCC correlates with tumor aggressiveness and poor prognosis.^[15]^ CHKA-mediated increases in intracellular phosphatidylcholine levels promote cellular growth and survival, driving cancer progression.^[27,28]^ These findings highlight the complex interplay between lipid metabolism and tumorigenesis in liver cancer, where dysregulation of cholesterol and lipid biosynthesis contributes to malignant transformation and tumor progression.

Our study also assessed the prognostic significance of FABP5 and ADH4, 2 key cholesterol-related genes, using univariate and multivariate Cox regression analyses. FABP5, a member of the FABPs which play a central role in cancer initiation and progression through the regulation of metabolic pathways, signal transduction, and gene expression, was considered as a diagnostic biomarkers and therapeutic targets in cancers.^[29]^ Consistent with previous reports, we found that FABP5 was upregulated in HCC tissues, and its high expression was associated with poor prognosis and higher recurrence rates.^[30–32]^ FABP5 promotes tumorigenesis by altering lipid metabolism and facilitating epithelial–mesenchymal transition – a crucial process in cancer metastasis.^[30,32]^ Through its interaction with the IL-6/STAT3/VEGFA signaling pathways, FABP5 enhances tumor progression by facilitating the migration and invasion of HCC cells.^[32]^ These results reinforce the importance of FABP5 in regulating intracellular cholesterol and lipid homeostasis, influencing tumor behavior.

In contrast, ADH4, a member of the alcohol dehydrogenase family, exhibited lower expression in liver cancer tissues and has been identified as an independent prognostic factor for improved survival. This inverse relationship between ADH4 expression and HCC progression suggests a protective role of ADH4 in liver carcinogenesis. Previous studies have suggested that ADH4 may counteract the oncogenic effects of altered cholesterol metabolism by modulating oxidative stress and reducing inflammatory signaling, thus slowing tumor growth.^[17,33]^ Our findings further support the hypothesis that ADH4 acts as a negative regulator of liver cancer progression, highlighting its potential as a therapeutic target.

The prognostic model developed in this study, incorporating FABP5 and ADH4 expression levels and clinical information age and TNM stage, demonstrated a C-index of 0.683, offering a novel tool for predicting patient outcomes in HCC. To our knowledge, the prognostic model constructed using clinical information and 2 survival related cholesterol homeostasis gene – FABP5 and ADH4 – is the first beneficial attempt to unveil the mechanism of liver cancer from the perspective of cholesterol Homeostasis, which may provide new ideas for the treatment and prevention of the disease. However, while this model shows promising results, results from the ICGC population were not completely consistent (Figs. S1–S3) and further validation in larger prospective cohorts is still necessary. Additionally, future studies should explore the influence of other factors, such as genetic mutations, environmental exposures, and drug interactions, on cholesterol homeostasis and their potential impact on HCC prognosis.

In conclusion, our study identified FABP5 and ADH4 as key prognostic markers in HCC and developed a novel prognostic model based on cholesterol homeostasis genes. This model offers important insights into the role of cholesterol metabolism in HCC progression and may guide personalized treatment strategies for HCC patients. Given the growing recognition of the role of cholesterol in cancer biology, FABP5 and ADH4 are promising targets for therapeutic intervention in liver cancer, warranting further investigation of their potential as biomarkers and therapeutic agents.

## Acknowledgments

We wish to thank all authors for their useful suggestions.

## Author contributions

**Conceptualization:** Jianghua Huo, Dan Meng.

**Data curation:** Chongyu Ding, Jianghua Huo, Ya-Qian Xu, Hui Zhang, Dan Meng.

**Formal analysis:** Chongyu Ding, Yulu Gong, Darong Hao.

**Methodology:** Jianghua Huo.

**Supervision:** Chongyu Ding.

**Writing – original draft:** Chongyu Ding, Jianghua Huo, Dan Meng.

**Writing – review & editing:** Chongyu Ding, Jianghua Huo, Ya-Qian Xu, Hui Zhang, Yulu Gong, Darong Hao, Dan Meng.












