# Supplementary material for: Expression and prognostic value of cholesterol homeostasis genes in hepatocellular carcinoma: A cohort study based on TCGA
Source: Medicine (Baltimore). 2026 May 22;105(21):e48547. doi: 10.1097/MD.0000000000048547 (PMC13200945; doi:10.1097/MD.0000000000048547)
Supplement: Supplementary file 1 [file medi-105-e48547-s001.docx]

**Supplementary Table 1. The detail clinical characteristics of selected subjects from TCGA.**

| characteristics | Overall(N=370) |
| --- | --- |
| Sex, n(%) |  |
| Male | 249 (67.3) |
| Female | 121 (32.7) |
| Age (mean (SD)) | 59.44 (13.52) |
| TNM Stage, n(%) |  |
| Ⅰ+Ⅱ | 256 (69.2) |
| Ⅲ+Ⅳ | 90 (24.3) |
| NA | 24 ( 6.5) |
| WHO Grade, n(%) |  |
| G1+G2 | 232 (62.7) |
| G3+G4 | 133 (35.9) |
| NA | 5 ( 1.4) |

SD, standard deviation; TNM, tumor-node metastasis.
