# Supplementary material for: Expression and prognostic value of cholesterol homeostasis genes in hepatocellular carcinoma: A cohort study based on TCGA
Source: Medicine (Baltimore). 2026 May 22;105(21):e48547. doi: 10.1097/MD.0000000000048547 (PMC13200945; doi:10.1097/MD.0000000000048547)
Supplement: Supplementary file 2 [file medi-105-e48547-s002.docx]

**Supplementary Table 2. the results of full model and stepwise model in TCGA.**

| Variable | full model | | stepwise model | |
| --- | --- | --- | --- | --- |
|  | β | RR | β | RR |
| Age | 0.0107 | 1.011(0.997-1.025) | 0.0114^·^ | 1.012(0.998-1.025) |
| Sex (male) | -0.0785 | 0.925(0.638-1.339) |  |  |
| TNM stage  (T3+T4) | 0.9416^***^ | 2.564(1.794-3.665) | 0.9407^***^ | 2.562(1.793-3.661) |
| WHO grade (G3+G4) | -0.0788 | 0.924(0.627-1.363) |  |  |
| FABP5 | 0.2361^*^ | 1.266(1.042-1.539) | 0.2235^*^ | 1.250(1.036-1.509) |
| ADH4 | -0.0885^**^ | 0.915(0.861-0.973) | -0.0884^**^ | 0.915(0.862-0.972) |

RR, risk ratio; TNM, tumor-node metastasis.

^·^p< 0.1, ^*^ p<0.05, ^**^ p<0.01,^***^ p<0.001.
