# Supplementary material for: Expression and prognostic value of cholesterol homeostasis genes in hepatocellular carcinoma: A cohort study based on TCGA
Source: Medicine (Baltimore). 2026 May 22;105(21):e48547. doi: 10.1097/MD.0000000000048547 (PMC13200945; doi:10.1097/MD.0000000000048547)
Supplement: Supplementary file 3 [file medi-105-e48547-s003.docx]

**Supplementary Table 3. the fit performance of two prognositc prediction models in TCGA.**

| Model | AIC | BIC | Log-Likelihood | C-index |
| --- | --- | --- | --- | --- |
| Full Model | 1251.24 | 1268.26 | -619.62 | 0.680 |
| Stepwise Model | 1247.55 | 1258.90 | -619.78 | 0.683 |

AIC, Akaike Information Criterion; BIC, Bayesian Information Criterion; C-index, Concordance Index.
