# Supplementary figures and images for: Expression and prognostic value of cholesterol homeostasis genes in hepatocellular carcinoma: A cohort study based on TCGA
Source: Medicine (Baltimore). 2026 May 22;105(21):e48547. doi: 10.1097/MD.0000000000048547 (PMC13200945; doi:10.1097/MD.0000000000048547)

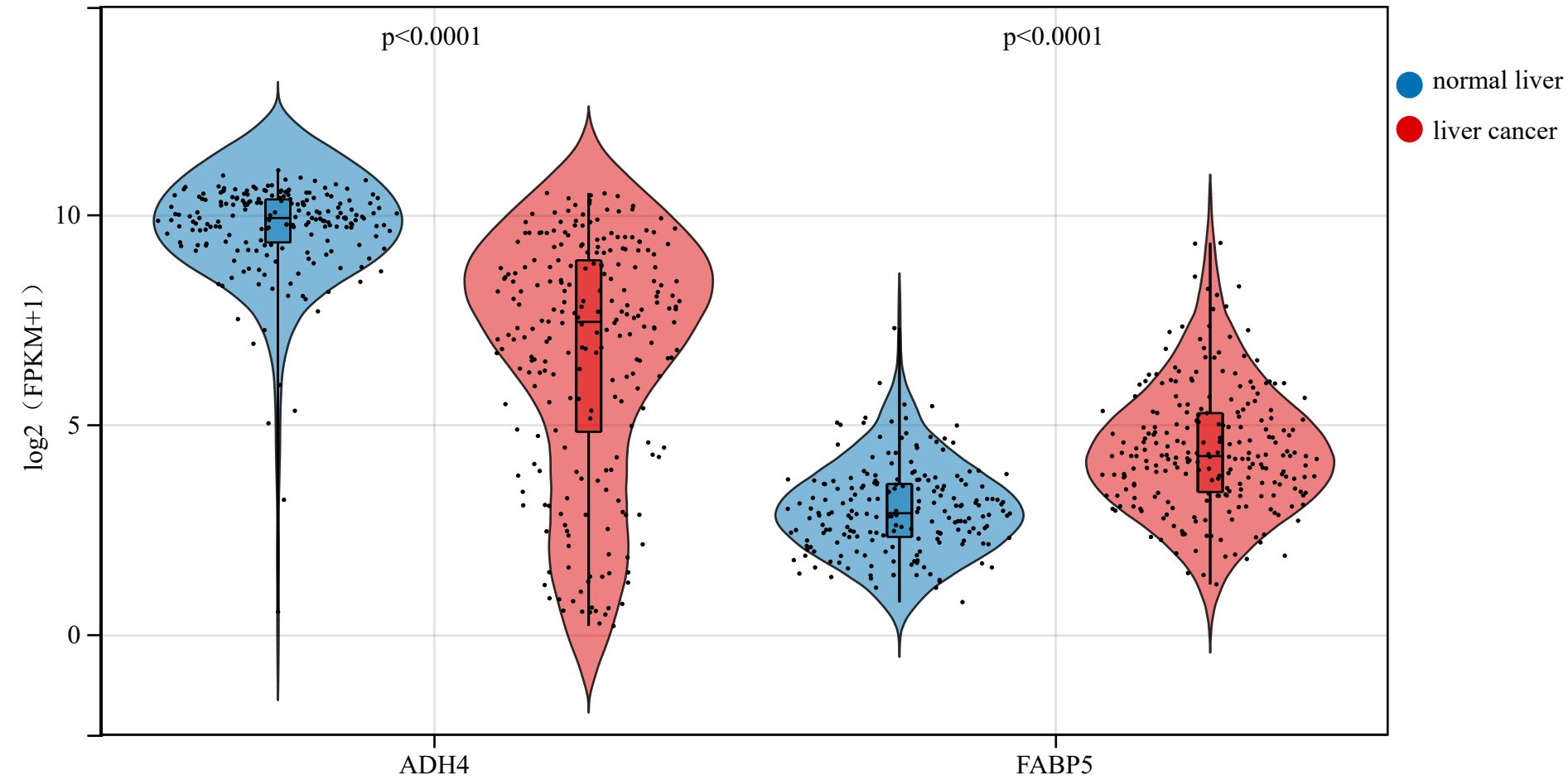

Supplement: Supplementary file 4 [file medi-105-e48547-s004.pdf]

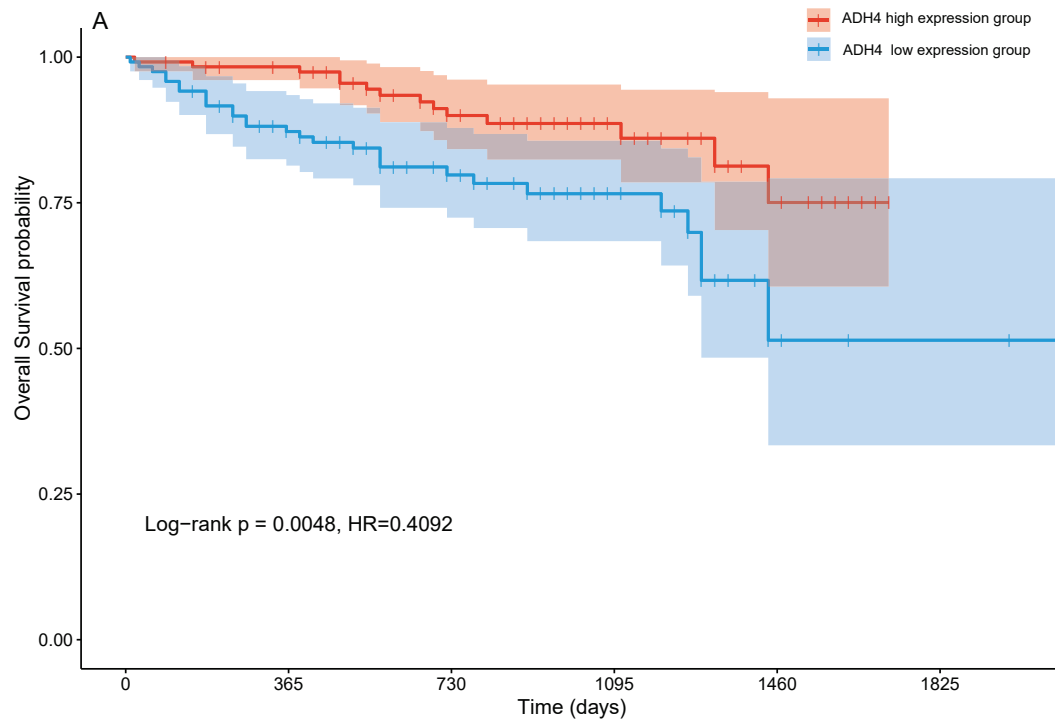

Number at risk

120 112 69 35 12 0

120 95 57 27 4 2

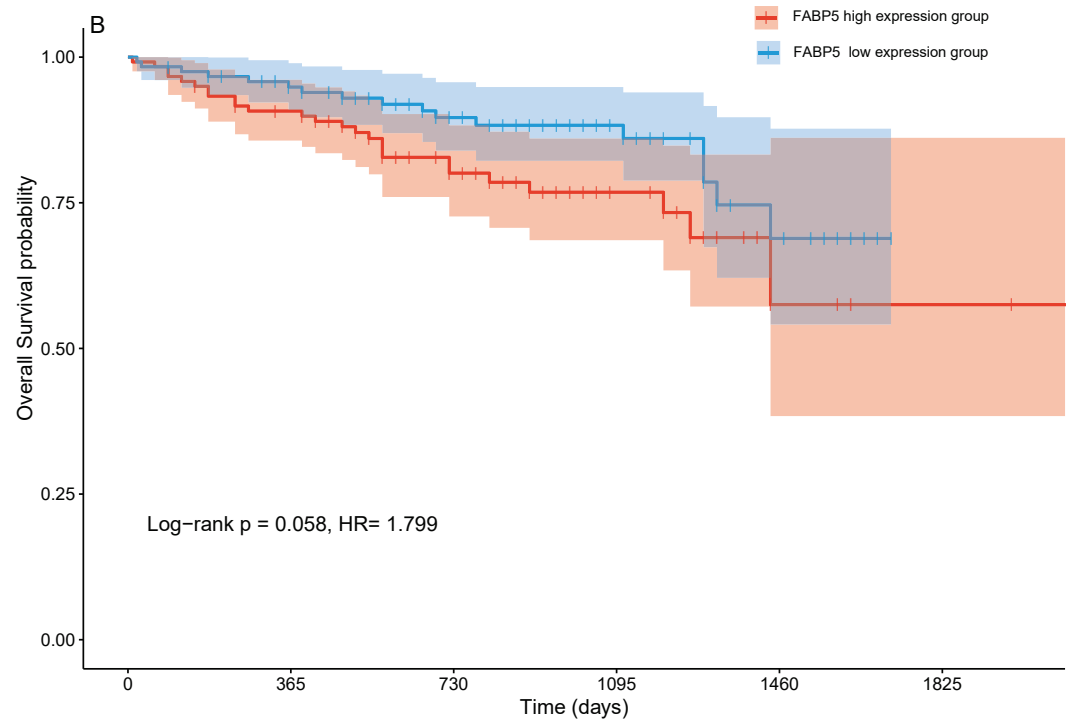

Number at risk

120 104 56 23 4 2

120 103 70 39 12 0

Supplement: Supplementary file 5 [file medi-105-e48547-s005.pdf]

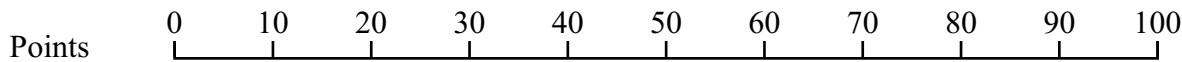

FABP5(p=0.98)

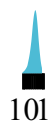

ADH4(p<0.05)

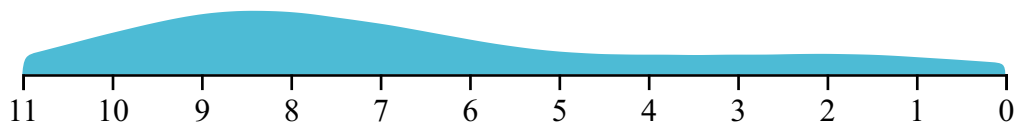

age(p=0.47)

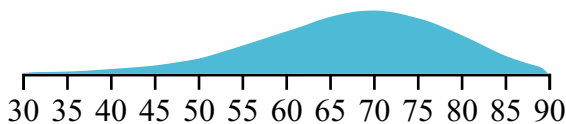

TNM stage(p<0.05)

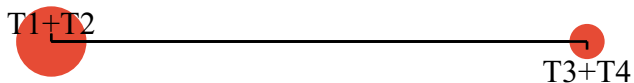

Total points

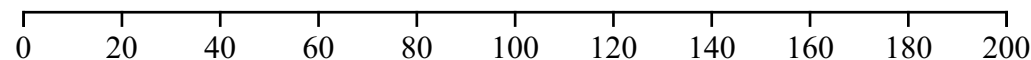

Linear Predictor

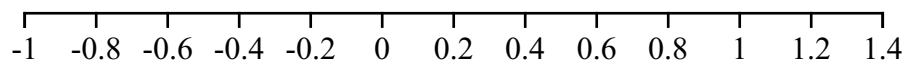

1-year OS

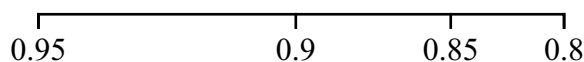

2-year OS

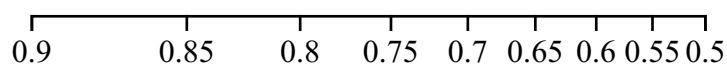

3-year OS

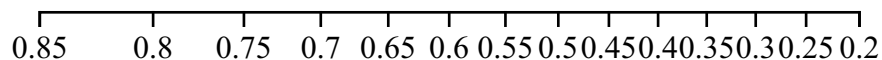

Supplement: Supplementary file 6 [file medi-105-e48547-s006.pdf]
